# Nootropic foods in neurodegenerative diseases: mechanisms, challenges, and future

**DOI:** 10.1186/s40035-025-00476-7

**Published:** 2025-04-03

**Authors:** Le Anh Minh Nguyen, Courtney Wayne Simons, Raymond Thomas

**Affiliations:** 1 Biology Department, Biotron Experimental Climate Change Research Centre, Western University, London, ON N6A 3K7 Canada; 2 Wright State University-Lake Campus, 7600 Lake Campus Drive, Celina, OH 45822 USA

**Keywords:** Neurodegenerative diseases, Environmental stressors, Neuroprotective foods, Nootropic foods, Diets

## Abstract

Neurodegenerative diseases (NDDs) such as Alzheimer’s and Parkinson’s disease are increasing globally and represent a significant cause of age-related death in the population. Recent studies emphasize the strong association between environmental stressors, particularly dietary factors, and brain health and neurodegeneration unsatisfactory outcomes. Despite ongoing efforts, the efficiency of current treatments for NDDs remains wanting. Considering this, nootropic foods with neuroprotective effects are of high interest as part of a possible long-term therapeutic strategy to improve brain health and alleviate NDDs. However, since it is a new and emerging area in food and neuroscience, there is limited information on mechanisms and challenges to consider for this to be a successful intervention. Here, we seek to address these gaps by presenting a comprehensive review of possible pathways or mechanisms including mutual interactions governing nootropic food metabolism, linkages of the pathways with NDDs, intake, and neuroprotective properties of nootropic foods. We also discuss in-depth intervention with nootropic compounds and dietary patterns in NDDs, providing a detailed exploration of their mechanisms of action. Additionally, we analyze the demand, challenges, and future directions for successful development of nootropic foods targeting NDDs.

**Supplementary Information:**

The online version contains supplementary material available at 10.1186/s40035-025-00476-7.

## Introduction

### Overview of neurodegenerative diseases (NDDs)

NDDs refer to a group of pathological conditions that adversely impact the central nervous system. These diseases are characterized by progressive degeneration and functional loss of neurons over time, leading to eventual death [1, 2]. In recent years, the incidence of NDDs and other brain health impairments has been on the rise and is anticipated to continue increasing in the future [3]. The upward trend poses a significant challenge to human health, society, and the economy. NDDs are responsible for 9 million deaths annually, making them a leading cause of death worldwide [4]. Following the COVID-19 pandemic, numerous reports have documented a general decline in brain health post-recovery, including neurological sequelae [5–7].

The most prevalent NDDs are Alzheimer’s disease (AD) and Parkinson's disease (PD), followed closely by Huntington’s disease (HD) and amyotrophic lateral sclerosis (ALS) [2]. Among the estimated 55 million individuals globally affected by dementia, over 30 million have AD [8]. NDDs account for 50% of total care costs. The annual expenses for older adults with AD are 4 times higher than those without AD. Projections indicate that these costs will double by 2050, reaching upwards of 140,000 USD per capita. The expansion of medical and caregiving expenses with these conditions places a substantial burden on both patients and the economy [8, 9].

Efforts to explore the etiology and treatments for NDDs have yielded certain advances [2, 10]. Current treatments are mainly centered on targeted immunotherapy. They aim to address specific NDD symptoms or biomarkers to control disease progression. Conventional pharmaceutical approaches involve blocking inflammatory mediators such as interleukins (IL) or tumor necrosis factor-alpha (TNF-α). Others target the deposition of misfolded proteins such as amyloid beta (Aβ) or tau [11, 12]. Notably, promising outcomes have been observed with glucagon-like peptide-1 receptor (GLP1R) agonists. They demonstrate potential in reducing neuronal loss and mitigating behavioral impairment in a mouse model by impeding the formation of neurotoxic A1 astrocytes [13]. Recent attention has also been directed towards novel potential interventions, including protective gene variants such as amyloid precursor protein (*APP*) A673T to reduce Aβ, and Apolipoprotein E (*APOE*)- ε2 to reduce the risk of AD [14]. However, despite the potential of these efforts, effective treatments for NDDs remain elusive and a definitive cure remains undiscovered [2, 3, 14].

In the current context, preventive actions through practicing brain-healthy diets and nutrition are expected to mitigate age-related risk factors and offer effective, affordable, and safe solutions for promoting a healthy brain and preventing NDDs later in life.

### Importance of diet and nootropic foods

In addition to genetic factors, environmental factors such as diets, air pollution, and radiation are known to play a significant role in NDD development [2, 15]. A substantial portion of AD cases in the 40–50 age group have been linked to genetic and environmental factors [10]. Notably, a diet high in sugar and saturated fat may accelerate brain aging, is recognized as a natural precursor to NDD, and worsens NDD symptoms [16]. Furthermore, a 12-year clinical study reported that unhealthy dietary components like alcohol or artificial sweeteners lead to a decline in cognitive function in older adults. Conversely, consumption of polyphenol-rich fruits, vegetables, mushroom, and red wine as well as their metabolites appears to confer a protective effect against cognition decline [17].

**Fig. 1:**
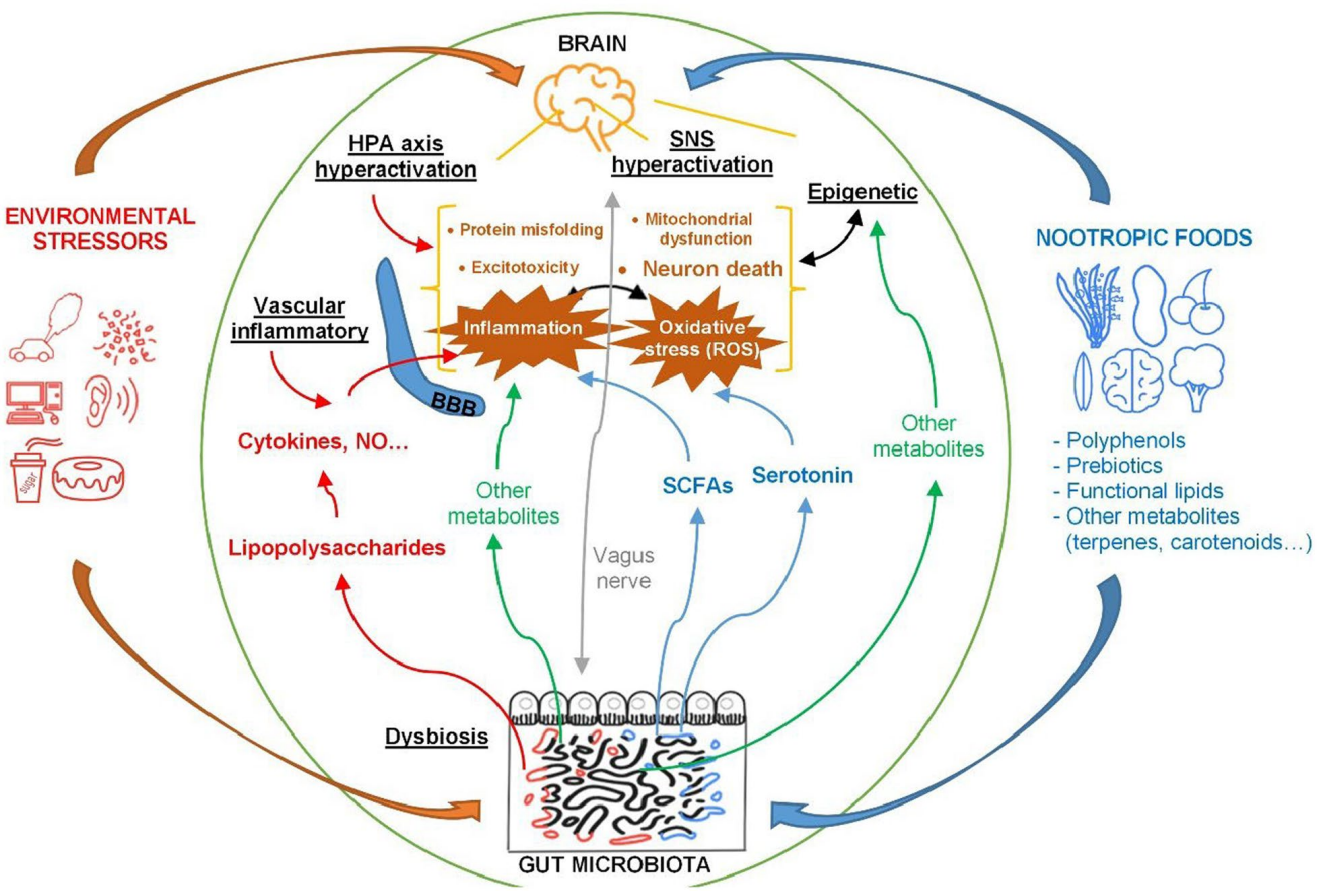
Effects of environmental stressors and nootropic foods on pathways leading to neurodegenerative diseases. HPA: hypothalamic–pituitary–adrenal axis; SNS: sympathetic nervous system; ROS: reactive oxygen species; BBB: blood–brain barrier; SCFAs: short-chain fatty acids

There is an intricate relationship between environmental stressors, including diets, and the development of NDDs (Fig. 1). For example, when exposed to environmental stressors, the hypothalamic–pituitary–adrenal axis and the sympathetic nervous system can be activated. This prompts a significant release of glucocorticoid and catecholamine hormones. Prolonged exposure to these hormones can result in chronic inflammation and oxidative stress within the brain [18]. This cascade of events is further accompanied by an increase in mediators like prostaglandins E2 and release of reactive oxygen species (ROS) during regulation of inflammation [19]. The impacts of environmental stressors also extend to the gut microbiota. They can lead to dysbiosis and subsequent secretion of lipopolysaccharides (LPS) as toxins, triggering an inflammatory response [20, 21]. Additionally, these stressors can influence epigenetic processes associated with activation of the inflammatory and oxidative stress pathways [22–24]. Oxidative stress and inflammation mutually sustain each other, spearheading the progression of neurodegeneration. The consequences of this intricate interplay include neuronal damage and death, mitochondrial dysfunction, and protein misfolding, all contributing to the development of NDDs [25–27].

Compared to the concept of neuroprotective foods that suggests the potential to improve brain function and mitigate NDDs [10, 28, 29], the new technical term, nootropic foods, is defined as neuroprotective foods specifically designed to enhance brain health-related cellular function, cognition, memory, and learning along the aging spectrum. Most nootropic foods are based on natural compounds such as vitamins and antioxidants or natural extracts from plants like *Ginko biloba* [30, 31]. Recent studies have delved into the efficacy of these foods on NDDs. As depicted in Fig. 1, the neuroprotective or nootropic compounds such as phytochemicals, prebiotics, and functional lipids, protect neurons from stressors, cell cycle progression, or death through scavenging ROS, reducing inflammation, and decreasing protein misfolding, mitochondrial dysfunction, or excitotoxicity in brain cells [25, 32]. They also balance gut microbe composition and modulate epigenetics processes, both of which are believed to be novel mechanisms in preventing and delaying the onset of NDDs [21, 33].

### Purpose and scope of the review

Nootropic foods containing bioactive compounds with neuroprotective potential have recently attracted global interest and research focus on preventing or managing NDDs [10, 34, 35]. However, existing research on food-related factors in NDDs occurs in isolation, lacking a comprehensive, interconnected map in the scientific literature. The development and validation of nootropic food products for NDDs is an emerging area, with very limited information on proven health benefits. In the absence of a comprehensive regulatory framework for nootropic foods, the risk of exaggerated marketing claims remains high. It is essential to approach these claims cautiously and ensure they are substantiated by robust scientific evidence. Acknowledging these challenges, this review provides details of the current knowledge surrounding the key metabolic pathways of NDDs implicated in foods and a comprehensive picture of the interactions. Additionally, we discuss the mechanisms of nootropic food intervention, emphasize the demand and challenges for further validation including translational application, and propose potential future directions for the development of nootropic products targeted at NDD.

We searched databases such as PubMed, Scopus, and Web of Science for research papers on nootropic foods, neuroprotective compounds, and their relationships to NDD, published in the recent 10 years (2014–2024). Primary keywords included “nootropic foods”, “nootropics”, “neuroprotective compounds”, “neurodegenerative diseases”, “neuroprotection”, “Alzheimer’s”, “Parkinson’s”, “amyotrophic lateral sclerosis”, “Huntington’s”, “dietary patterns”, “dietary intervention”, and “nutritional supplementation”. Search strings combined the keywords such as: “nootropic foods” or “nootropics” and “neurodegenerative diseases”; “dietary intervention” and “neuroprotection”; “dietary intervention” and “neurodegenerative diseases”; “Alzheimer’s disease” and “dietary intervention”; “amyotrophic lateral sclerosis” and “neuroprotective foods”. We included peer-reviewed original studies with detailed methodology, including bioassays or clinical trials that explored potential mechanisms underlying neuroprotection. Studies with comprehensive results and discussions in the management of NDDs with neuroprotective compounds or diets were prioritized. Articles that were non-peer-reviewed, non-English, retracted, or lacking full-text access were excluded. Other types of papers such as reviews, book chapters, editorials, commentaries, conference abstracts, letters to the editor, and opinion pieces were also excluded. Further exclusion criteria included studies lacking detailed methodologies, those without bioassays to reveal neuroprotective mechanisms, and those involving participants with severe metabolic conditions or serious medical conditions such as death during treatment (Fig. S1). To complement the literature, we also referred the most recent relevant reports from esteemed organizations such as the World Health Organization and the National Institute of Health of the United States to incorporate global statistics and context on NDD.

## Primary NDD pathways associated with diets and nootropic food interventions

Diets and other environmental factors have been observed to contribute to NDDs primarily through the following five pathways: (1) oxidative stress via elevated ROS levels [18], (2) mitochondrial dysfunction [36], (3) formation of misfolded protein plaques [32], (4) chronic neuroinflammation [37], and (5) excitotoxicity [38]. These pathways ultimately cause neuronal cell injury or death via apoptosis or autophagy, resulting in the malfunction of neurons and the onset of NDDs.

**Fig. 2:**
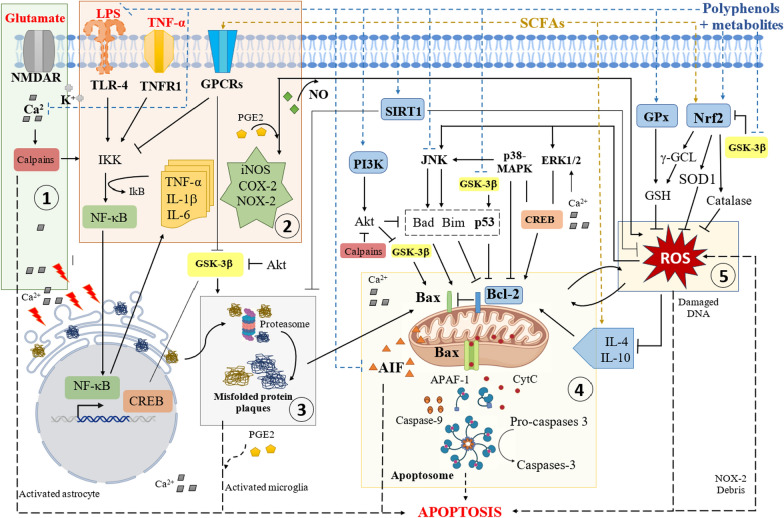
Major pathways of neurodegenerative diseases that are closely related to environmental stressors and food metabolites. The five major pathways of NDDs include: (1) excitotoxicity; (2) neuro-inflammation; (3) formation of misfolded protein plaques; (4) mitochondrial dysfunction; and (5) oxidative stress. Functional components such as polyphenols and short-chain fatty acids (SCFAs) can reverse or prevent these pathways. NMDAR: *N*-methyl *D*-aspartate receptor; TLR-4: toll-like receptor 4; TNFR1: tumor necrosis factor receptor 1; GPCRs: G-protein-coupled receptors; IKK: IkappaB kinase; NF-κB: nuclear factor kappa B; TNF-α: tumor necrosis factor-alpha; IL: interleukin; PGE_2_: prostaglandin E2; NO: nitric oxide; iNOS: inducible nitric oxide synthase; COX-2: cyclooxygenase 2; NOX-2: nicotinamide adenine dinucleotide phosphate (NADPH) oxidase 2; GSK-3β: glycogen synthase kinase 3 beta; CREB: cAMP (cyclic adenosine 3′,5′-monophosphate) response element-binding protein; SIRT1: sirtuin 1; PI3K: phosphoinositide-3-kinase; Akt: protein kinase B; JNK: c-Jun-N terminal kinase; p38-MAPK: p38 mitogen-activated protein kinase; p53: transcription factor p53; ERK1/2: extracellular signal-regulated kinase ½; Bcl2: protein B cell lymphoma 2; AIF: apoptosis-inducing factor; CytC: cytochrome c; GPx: glutathione peroxidase; GSH: glutathione; Nrf2: nuclear factor erythroid-2-related factor 2; γ-GCL: gamma-glutamyl cysteine ligase; SOD1: superoxide dismutase 1

The five pathways are interconnected and strongly associated with chronic inflammation and oxidative stress in the brain (Fig. 2) [39, 40]. Certain nootropic compounds such as short-chain fatty acids (SCFAs) and polyphenols may delay or block the activation of these metabolic pathways with consequential effects, facilitating recovery of damaged neurons [1, 40, 41].

**Fig. 3:**
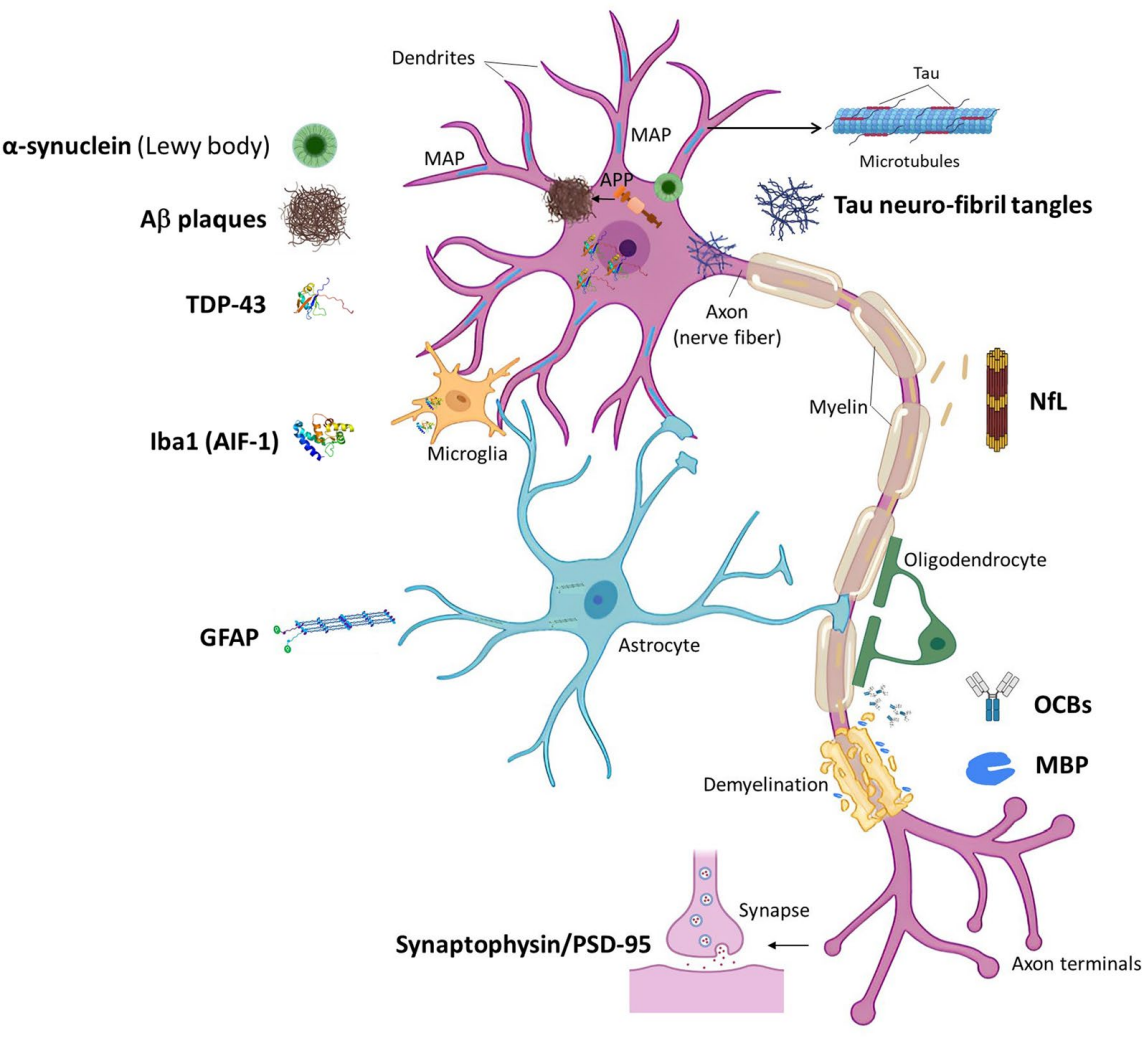
Some specific biomarkers for neurodegenerative diseases. Aβ: Amyloid beta; TDP-43: TAR-DNA-binding protein 43; Iba1: ionized calcium-binding adaptor molecule 1; GFAP: glial fibrillary acidic protein; PSD-95: postsynaptic density protein 95; NfL: neurofilament light chain; OCBs: oligoclonal bands; MBP: myelin basic protein

Damaged neurons release specific biomarkers into cerebrospinal fluid and blood, which can indicate the NDD status during diagnosis and treatment. These biomarkers include transactive response-DNA-binding protein 43 (TDP-43), neurofilament light chain (NfL) protein, and myelin basic protein (MBP) (Fig. 3). TDP-43 undergoes mislocalization from the nucleus to the cytoplasm in damaged neuronal cells. NfL and MBP are produced due to demyelination [42–44]. Elevated levels of these biomarkers are associated with increased severity of NDDs.

Interestingly, beyond effects on the five primary pathways, recent studies suggest that certain nootropic compounds may also promote axon regeneration and adult neurogenesis, offering additional therapeutic potential for NDDs. Adult neurogenesis, the formation of new neurons in the adult brain, particularly in the hippocampus, may be stimulated by dietary components such as flavonoids, resveratrol, and polyunsaturated acids (PUFAs) [45, 46]. This process is thought to be mediated by the expression of neurotrophic factors such as cAMP response element binding protein (CREB) or brain-derived neurotrophic factor (BDNF), which may enhance neuronal growth through activation of the Wnt/β-catenin pathway via GSK-3β (glycogen synthase kinase 3 beta) [47, 48].

Similarly, axon regeneration, a critical process for repairing damaged neuronal fibers, has been partially linked to bioactive compounds like Astragaloside VI, a saponin extracted from *Astragalus membranaceus*, or Hericene A, an isoindoline from *Hericium erinaceus* mushroom. They may exert their effects partly through tropomyosin receptor kinase B (TrkB), a receptor for BDNF [49, 50]. This emerging evidence underscores the multifaceted neuroprotective capabilities of nootropic foods, extending their ability beyond combating neuron damage to actively supporting neuronal repair and regeneration. However, many relevant underlying mechanisms have not been discovered, emphasizing the need for further research to fully elucidate their roles and optimize their therapeutic applications.

### Excitotoxicity

Excitotoxicity, caused by abnormally high levels of amino acids such as glutamate within the synapse, plays a pivotal role in NDDs [38]. Elevated glutamate levels may stem from various environmental stressors such as a high-fat diet or exposure to exogenous glutamate such as dietary monosodium glutamate. While glutamate is an essential neurotransmitter in the brain, disruptions of its homeostasis can lead to excitotoxicity, ultimately resulting in neuron damage [51]. Excitotoxic neurodegeneration is closely associated with over-activation of the *N*-methyl *D*-aspartate receptor (NMDAR), which can lead to an excessive influx of calcium (Ca^2+^) and trigger activation of pro-apoptotic proteins such as calpains and p53, resulting in neuronal injury and apoptosis [52]. In the context of HD, increased NMDAR activation is linked to the mutations of the *HTT* gene and the expression of postsynaptic density protein 95 [38] (Fig. 3). A recent study identified AD-specific transcriptomic signatures in glutamate-injured HT22 cells, further linking glutamate-induced neurotoxicity to AD [53]. Additionally, neuronal damage caused by excitotoxicity in NDDs can be detected early through increased levels of S100β, a calcium-binding protein released by glial cells in response to acute kainate or glutamate injury in the brain [54].

Excitotoxicity is intricately connected to other pathological pathways in NDD. Elevated levels of Ca^2+^ can cause endoplasmic reticulum (ER) stress, which is associated with protein misfolding and mitochondrial dysfunction [53, 55]. Calpains contribute to the activation of the nuclear factor kappa B (NF-κB) pathway via IkappaB (IkB) kinase (IKK) [56]. The NF-κB pathway generates pro-inflammatory cytokines such as TNF-α, IL-1β, and IL-6 [57, 58], and upregulates the levels of oxidative enzymes, including inducible nitric oxide synthase (iNOS), cyclooxygenase 2 (COX-2), and nicotinamide adenine dinucleotide phosphate (NADPH) oxidase 2 (NOX-2). Importantly, these enzymes contribute to the induction of oxidative stress, thereby exacerbating neuronal damage [59–61].

### Chronic neuroinflammation

Microglia and astrocytes play pivotal roles in protecting neuronal cells against various harmful factors by producing pro-inflammatory or anti-inflammatory cytokines and enzymes to regulate neuroinflammatory responses [62, 63]. However, chronic or excessive neuroinflammation, whether resulting from aging or triggered by environmental stressors, is considered a major pathology of NDDs [37, 39]. In patients with AD and PD, glial fibrillary acidic protein (GFAP) released from active astrocytes and ionized calcium-binding adaptor molecule 1 (Iba1) from active microglia under neuroinflammation are associated with loss of myelin and increased soluble tau level in neurons [63]. Specific markers of overwhelmed immune system activity in the central nervous system have been identified, such as oligoclonal bands (OCBs), which are immunoglobin bands produced by B cells infiltrating the brain [64] (Fig. 3). Diets and other environmental stressors can induce neuroinflammation in NDDs through various mechanisms. For instance, the expression of calpains induced by excitotoxicity can trigger the IKK/NF-κB pathway, leading to neurodegeneration. Gut dysbiosis associated with unhealthy diets such as high-fat or low-fiber diets, can lead to a leaky gut and secretion of LPS. In turn, LPS may activate toll-like receptor-4 (TLR-4), subsequently triggering the IKK/NF-κB pathway [57, 62]. The translocation of the NF-κB pathway followed by production of cytokines and relevant enzymes such as iNOS, COX-2, and NOX-2, is a key response in neuroinflammation [58, 65, 66]. Another noteworthy mechanism involves epigenetic processes, such as the over-activation of histone deacetylases, leading to deactivation of SIRT1, a pro-survival and anti-inflammatory protein. This, in turn, contributes to the neuroinflammation observed in NDDs [10, 67].

Neuroinflammation and oxidative stress are closely interrelated, with each capable of sustaining and intensifying the other. ROS stimulate transcription factors such as NF-κB and activator protein 1 (AP-1), resulting in the upregulation of pro-inflammatory genes [37]. Conversely, nitric oxide (NO) and prostaglandin E_2_ (PGE_2_) produced by iNOS and COX-2 during neuroinflammation, contribute to increased ROS levels [68]. In addition, neuroinflammation is closely linked to mitochondrial dysfunction via mitogen-activated protein kinase (MAPK) and c-Jun-N terminal kinase (JNK) pathways. For example, TNF-α released from peripheral cells promotes these pathways, leading to activation of tumor necrosis factor receptor 1 (TNFR1). This subsequently leads to the activation of the IKK/NF-κB pathway, further contributing to neuronal damage [58, 69].

### Formation of misfolded protein plaques

The accumulation of misfolded protein plaques of tau and Aβ, as well as α-synuclein, are prominent features of AD and PD, respectively. These toxic structures significantly impair neuronal function and induce degeneration and death of neurons [53, 70, 71]. The presence of misfolded proteins is often associated with ER stress caused by exposure to abnormal glutamate exocytosis or high ion influx under excitotoxic conditions [53, 55]. In addition, excessive activation of beta-site APP cleaving enzyme 1 and glycogen synthase kinase 3 beta (GSK-3β) can lead to increased production of Aβ and hyper-phosphorylation of tau protein, respectively, thereby fostering their misfolding and aggregation [32, 72] (Fig. 3). Dietary interventions with neuroprotective or nootropic foods can exert protective effects on these mechanisms [32, 70, 73]. For example, polyhydroxy curcuminoids, tovophyllin A from mangosteen or Western diet can modulate levels of APP or neprilysin (NEP) methylation to control Aβ degradation or modulate GSK-3β overexpression [74–76]. Cerebrospinal fluid levels of AD biomarkers, such as Aβ42, Aβ40, p-tau and total tau, are associated with the amounts and the types of PUFAs in the diet. High levels of PUFAs (60%–65%) from olive oils during intervention with modified Mediterranean-ketogenic diet increase soluble Aβ42 and decrease tau [77, 78].

Misfolded protein aggregation is implicated in oxidative stress and mitochondrial dysfunction [70]. In neuronal cells, Aβ-induced toxicity is linked to increased oxidative stress, marked by elevated levels of ROS as well as increased activity and expression of NOX [79]. Interestingly, recent findings propose that low levels of Aβ might reduce oxidative stress while enhancing the function of reductase enzymes superoxide oxide dismutase (SOD) and glutathione peroxidase [80]. Aβ42 plaques affect mitochondrial function through increased phosphorylation of extracellular signal-regulated kinase (ERK1/2) and p38 MAPK, as well as reduced ratio of phosphorylated CREB/total CREB in SH-SY5Y cells [79]. Aβ42 deposition also decreases the level of antiapoptotic protein B cell lymphoma 2 (Bcl-2), upregulates the pro-apoptotic protein Bax, and increases activities of apoptosis-related proteins caspase-3, caspase-9 and cytochrome c (Cytc) in SH-SY5Y cells, ultimately causing apoptosis and neurite degeneration [70, 79, 81].

### Mitochondrial dysfunction

Mitochondria in neurons are responsible for fueling neuronal cells and regulating a myriad of molecular mechanisms, including calcium modulation, lipid metabolism, and intracellular ROS production [36, 82]. Their dysfunction can lead to neuronal cell death through release of pro-apoptotic signaling proteins such as AIF (apoptosis-inducing factor), caspases, and CytC. Caspase-9, CytC and APAF-1 (apoptosis protease activating factor 1) can subsequently form apoptosomes, which then activate caspase-3 to trigger apoptosis [1, 52, 79]. Therefore, mitochondrial dysfunction is strongly implicated in NDDs such as AD, PD, HD, and ALS [36, 82]. For example, mutations of *PINK1* (PTEN-induced kinase 1), a key regulator of mitochondrial function and structure, have been linked to AD and PD [36]. Mutant huntingtin protein causes mitochondrial dysfunction through transcription factor p53 and peroxisome proliferator-activated receptor-gamma coactivator 1α (PGC-1α), a mitochondrial biogenesis factor. In NDDs, mitochondrial dysfunction is closely intertwined with diets and other environmental stressors, mediated by transcription factors such as heat shock response factors [83]. The transcription factor p53 can be modulated by diets and environmental factors via a network of SIRT, AMP-activated protein kinase (AMPK), MAPK, and CREB. For example, caloric restriction diet increases the levels of SIRT3, which activates CREB and AMPK, followed by PGC-1α stimulation and altered PINK levels [67, 84, 85]. Other transcription factors such as GSK-3β, p38-MAPK, ERK, Akt, JNK, and phosphoinositide-3-kinase (PI3K)/Akt have also been reported to mediate the neuroprotective effects of nootropic foods via their downstream factors like p53, Bax, or Bcl2 [56, 79, 83, 86–88] (Fig. 2).

Mitochondrial dysfunction often coincides with misfolded protein deposition, ER stress excitotoxicity, neuroinflammation, and oxidative stress. ROS, which are generated partly from the electron transport chain of the mitochondria, exacerbate oxidative stress [82, 86, 89] and in turn contribute to the malfunction of mitochondria.

### Oxidative stress

Oxidative stress, characterized by high levels of ROS in nerve cells, is a key cytotoxic event leading to neuronal cell death in NDDs [67, 91]. ROS can cause damage to various cellular biomolecules such as carbohydrates, lipids, proteins, and DNA in brain cells [27]. In NDD, ROS promote pro-apoptotic MAPK, ERK, and JNK pathways, while inhibiting the pro-survival PI3K/Akt pathway [25, 56, 86]. The most direct consequence is the depletion of antioxidants like glutathione (GSH), antioxidant enzymes including glutathione peroxidase, gamma-glutamylcysteine ligase (γ-GCL), and SOD1, or related proteins such as nuclear factor erythroid-2-related factor 2 (Nrf2) [26, 61, 68, 90, 91] (Fig. 2). Increased ROS levels may result from excitotoxicity, mitochondrial oxidation, misfolded protein aggregation, and neuroinflammation in NDDs [19, 59, 61, 79].

Oxidative stress is closely linked to diet and environmental stress [18]. For example, high-lipid diets increase the susceptibility to ROS-induced brain damage [67]. Notably, extracellular dietary compounds such as high contents of fat and heavy metals serve as a main source of well-known ROS, such as hydroxyl peroxide (H_2_O_2_), peroxyl radicals (OH•), superoxide anion (O_2_^−^), and peroxy-nitrite (ONOO^−^) [27, 68]. Conversely, a diet rich in antioxidants, fruits, vegetables, and anti-inflammatory agents can effectively mitigate ROS and modulate NDD pathways through Akt/NF-κB, TrkB/CREB/BDNF, Keap1 (Kelch-like erythroid cell-derived protein with cap ‘n’ collar (CNC) homology-associated protein 1)/Nrf2, or AMPK pathway [61, 65, 87, 90, 92, 93]. Specific phytochemicals such as stigmasterol have been identified to protect neurons against oxidative stress by enhancing the expression of SIRT1 and Bcl-2 [67].

## Nootropic compounds and their protection against NDDs

### Overview of nootropic compounds and their mechanisms

The close link between primary NDD pathways and dietary factors suggests a potential for nootropic foods to manage, prevent, or delay NDDs [10, 17, 29, 93, 94]. For example, anthocyanins with known nootropic properties have demonstrated protection of neuronal cells from oxidative stress, mitochondrial dysfunction, and excitotoxicity (Fig. 2) [59–61]. Similarly, β-glucan or (− )-epigallocatechin-3-gallate (EGCG) delays the deposition of misfolded proteins [73, 95]. A diet abundant in omega 3 (n-3) PUFAs can reduce the levels of endotoxins such as IL-6 and LPS [96]. Extracts rich in fibers or polyphenols can prevent activation of neuroinflammatory pathways and reduce expression of related markers [57, 70].

Nootropic compounds can exert their neuroprotective properties directly on molecular NDD pathways after absorption, or indirectly via epigenetic process or modulation of gut homeostasis. For example, certain food components such as Fe (II) and ascorbate can reduce levels of ROS radicals. They can also modulate epigenetic processes such as DNA methylation and histone acetylation by regulating the levels of hydroxyl radicals (OH^−^) [23]. Mao et al. further emphasized the role of dietary interventions in reshaping the metabolism–epigenetics–immunity cycle, thereby mitigating brain dysfunction associated with NDDs [33]. Modulation of epigenetic processes such as phosphorylation and methylation, by dietary factors, attenuates the primary pathways leading to NDDs [22, 24]. For instance, regulation of CREB phosphorylation levels can alter its activation and subsequently affect the expression of numerous downstream genes like *BCL2* and *BDNF* [79, 97, 98]. In addition, DNA hypermethylation of the gene encoding NEP, which controls Aβ degradation, reduces NEP expression, while hypomethylation of the APP gene increases its expression, both contributing to Aβ deposition [1, 10].

Recent hypotheses propose that NDDs may originate in peripheral systems, such as the gut. For example, PD has been proposed to arise from the gut through misfolding of α-synuclein in the enteric nervous system, which is consistent with clinical evidence that gut disorders often precede neurological signs [99]. Similarly, peripheral inflammation originating in the gut and spreading through the bloodstream has been identified as a potential risk factor for AD, correlating with elevated amyloid levels in AD dementia [100, 101]. These findings highlight the gut as a critical site of pathology and suggest that targeting gut health may represent a novel mechanism through which nootropic compounds mitigate NDD.

The gut microbiome is considered an important bridge between foods and NDDs based on the gut-brain axis [20, 102]. At a healthy state, the gut bacteria produce a wide range of bioactive compounds that can influence brain function and health through the gut-brain axis (Fig. 1) [20, 103]. Some gut microbes modulate the production of neurotransmitters, such as serotonin and dopamine [104]. Moreover, fermentation of dietary fiber by gut flora produces SCFAs, which may ameliorate neuroinflammation and protect neurons against the environmental stress [105, 106]. Conversely, imbalances in gut homeostasis, known as gut dysbiosis, are associated with metabolic disorders due to dietary habits or exposure to life stressors. These disturbances can trigger inflammation and increase the permeability of the blood–brain barrier (BBB) (Fig. 1) [39]. Gut dysbiosis may also impact intestinal permeability, leading to the release of toxins (such as LPS) into cells, including neurons [20, 21]. Subsequently, LPS can activate the immune system to secrete NO and pro-inflammatory cytokines IL-1β, IL-2, or TNF-α, resulting in chronic neuro-inflammation (Fig. 2) [37, 102]. In addition, the increased production of pro-inflammatory cytokines induced by gut dysbiosis, potentially compromises the BBB integrity [19], leading to the entry of toxins, peripheral inflammatory compounds, and T cells into the brain. This can disrupt the neuroimmune system, cause damage to nerve cells, and impair functions associated with brain detoxification [107]. Nootropic foods, particularly those affecting the gut microbiota and SCFA production, offer potential therapeutic benefits in reducing these early pathological changes. By restoring the gut-brain axis homeostasis and lowering systemic inflammation, nootropic foods may serve as an innovative strategy to prevent or delay the progression of NDDs [21]. Below we discuss in depth the mechanisms of primary and secondary metabolites of nootropic foods that can be considered for formulating nootropic foods targeting NDDs.

### Primary nootropic metabolites

#### PUFAs

Polyunsaturated acids or PUFAs, especially n-3 and omega 6 (n-6), are essential components of nerve cell membranes that help maintain optimal nerve and brain functions [108]. Specific n-3 and n-6 PUFAs found in the brain include eicosapentaenoic acid (EPA, 20:5, n-3), docosahexaenoic acid (DHA, 22:6, n-3), and arachidonic acid (ARA, 20:4, n-6). Notably, ARA accounts for 5%–11% of the total phospholipids in the brain, while DHA accounts for 13%–22% [109]. The significance of these PUFAs extends to their role as precursors of eicosanoids such as PGE_3_, thromboxane A (ThBA) 3, and leukotrienes B (LTB) 5. These eicosanoids have potent anti-inflammatory effects [96, 110]. However, under aging or stress conditions, bioactive derivatives of ARA, such as GE2, ThBA2, and LTB4, may be upregulated, contributing to neuronal inflammation. In contrast, DHA‐derived mediators such as maresin 1, neuroprotectin D1, and resolvin D5, display marked anti-inflammatory and neuroprotective properties [111]. It is important to note that ARA and DHA cannot be interconverted within the body. Their levels are subject to alteration based on age and environmental factors, including diet [109]. This highlights the potential role of nutrition in modulating brain PUFA composition associated with NDD.

Dietary PUFAs are widely present in oily fish, nuts, green leafy vegetables, soybeans, seeds, and grains. The common n-3 and n-6 PUFAs present in foods are α-linolenic acid (ALA, 18:3, n-3) and linoleic acid (LA, 18:2, n-6), which are metabolized to DHA and ARA in the brain, respectively [96]. Recent studies have emphasized the importance of balanced levels of n-6 and n-3 PUFAs in the diet [15, 112]. Deficiency in n-3 PUFAs can increase the ARA/DHA ratio. Conversely, supplementation with DHA can reduce ARA levels in the cerebral cortex of rats [109]. Moreover, an excessive ratio of n-6:n-3 has been associated with both enhanced proliferation of the neuronal precursor cells and disrupted neurodevelopment in the early stage of life [112]. This high ratio also reduces the cell viability of microglia [15].

The mechanisms underlying these effects may involve excitotoxicity with increased expression of glutamate ionotropic receptors and gamma-aminobutyric acid receptors in synapses. This, in turn, can alter the gene expression of crucial neuronal markers such as microtubule-associated protein 2 (MAP2) and β-tubulin type III, as well as synaptic markers including PSD95 and synaptophysin [112]. The PUFA composition also impacts neuroinflammation by regulating cytokine secretion, particularly interleukins IL-4, IL-10, IL-17, and TNF-α [15]. Additionally, n-3 FAs can compete with ARA to reduce the production of pro-inflammatory eicosanoids like PGE_2_, ThBA2, and LTB4 [111]. The n-3 PUFAs can also reduce over-phosphorylation of tau protein and modulate gut microbiota [10]. Considering these compelling findings, a dietary approach or nootropic foods rich in n-3 PUFAs and surpassing n-6 PUFA concentration may be beneficial for the brain [96].

#### Amino acids and derivatives

Amino acids such as tryptophan, methionine, and arginine, are essential for brain function. Abnormalities in their metabolism can lead to brain disorders and NDDs [17].

Tryptophan is a precursor to serotonin, an important neurotransmitter regulating mood and behavior. Reduced levels of tryptophan can lead to increased formation of kynurenine, a metabolite of tryptophan, and are associated with neuropsychological symptoms. The elevated levels of kynurenine can over-activate NMDARs [113, 114] that are closely linked to excitotoxicity, neuro-inflammation, and NDDs (Fig. 2). In a rat model of AD, *N*-acetyl-*L*-tryptophan leads to downregulation of inflammatory markers such as TNF-α and IL-6 in the hippocampus and frontal cortex, and reduces acetylcholinesterase activity, NF-κB, and total and phosphorylated tau protein levels. It also upregulates CREB1 signaling, a pathway associated with neuroprotection and cognitive function [115].

Arginine is a precursor to NO. Arginine in the brain stimulates the production of cGMP, a second messenger molecule involved in various neuronal functions such as learning, memory, and immune responses. Disruption of arginine metabolism leads to altered immune response and NDDs [116–118].

Methionine and its metabolite homocysteine hold important implications for DNA and histone methylation. A high homocysteine/methionine ratio is found in patients with cognitive impairment and dementia and is also associated with enhanced expression of APP and decreased levels of NEP and IDE [119, 120].

Recent studies have shown that dietary intervention based on essential amino acids is a promising strategy for preventing and delaying NDDs. A diet supplement containing 7 essential amino acids (leucine, phenylalanine, lysine, isoleucine, histidine, valine, and tryptophan) can ameliorate brain atrophy and dementia in AD mice. The mechanisms may involve their ability to inhibit the blood-to-brain transfer of kynurenine, which is metabolized by glial cells in the brain. Gene Ontology analysis revealed that this supplement can reverse neuroinflammatory responses (*TNF-Α*, *Cox*1, and *Cox2*), regulate glial activation (*Gfap*), and reduce genetic AD risks (*Apoe*). Conversely, an essential protein-deficient diet can accelerate neurodegenerative processes [121]. In addition, administration of *L*-arginine can prevent neurodegeneration in PD mice induced by MPTP (1-methyl-4-phenyl-1,2,3,6-tetrahydropyridine) [118]. This intervention also reduces oxidative stress and apoptosis in PC-12 cells with Aβ amyloidosis, and improves spatial memory in AD mice [122].

Recent research in both in vitro and in vivo models has uncovered neuroprotective effects of an unessential amino acid, glycine, against *D*-galactose-induced NDD. Glycine inhibits oxidative stress, upregulates the expression of antioxidant protein Nrf2 and downregulates p-JNK expression. It also inhibits the *D*-galactose-induced apoptosis, mitochondrial dysfunction, and neuroinflammation [123]. In addition, a novel D-amino acid peptide that has been developed as an inhibitor of tau fibrillization, can potentially serve as a treatment for AD. In cell cultures, it prevents the cytotoxic effects of both externally added tau fibrils and internally expressed tau mutants [124].

#### SCFAs

SCFAs, including acetate (C2), propionate (C3), and butyrate (C4), are produced by gut microbes during dietary fiber fermentation. These compounds are known for their potential neuroprotective effects, particularly their anti-inflammatory properties [105]. SCFAs can inhibit histone deacetylase enzymes, allowing enhanced histone acetylation of genes for anti-inflammatory cytokines such as IL-4 and IL-10 [125]. Additionally, SCFAs act as ligands for G-protein-coupled receptors (GPCRs), including free fatty acid receptor 3 in neurons. Activation of GPCRs by SCFAs leads to the inhibition of the IKK/NF-κB pathway and subsequent decreased production of pro-inflammatory cytokines such as IL-6 and TNF-α. Moreover, GPCR activation triggers Nrf2, effectively reducing ROS levels in nerve cells [96, 105, 106, 126, 127]. Furthermore, SCFAs are likely to alleviate the hyperphosphorylation of tau protein in neurons following Aβ-induced injury [128]. They can also improve the gut-brain axis by increasing the production of neurotransmitters such as serotonin, thereby influencing brain function and behavior [106, 129].

While SCFAs are recognized to have neuroprotective properties, recent assessment indicates the need for further investigation [106, 130]. It is worth noting that only a small amount of SCFAs are actively metabolized by other organs. In the brain, the maximum concentrations of C2, C3, and C4 are about 171, 6, and 2.8 µmol/L, respectively. Interestingly, elevated levels of SCFAs in the circulatory system have been observed in patients with infectious and auto-inflammatory conditions. These heightened levels are linked to impaired neuronal functions [106, 131]. SCFAs at levels reported in human systemic circulation can adversely impact brain energy metabolism, impair neuro-lipid metabolism, and cause cell death [130]. Further research in this area is essential to fully understand the role of SCFA in mediating neuronal function, survival, and brain health outcomes.

#### Dietary fibers

Dietary fibers are crucial components of our diets and are found in a variety of sources such as fruits, vegetables, grains, and certain bacteria. They are indigestible carbohydrate polymers that are fermented only by the gut microbiome in the colon [108, 132, 133]. They have long been known as essential and health-promoting food ingredients, particularly as prebiotics. Actions of prebiotics are associated with gut-flora modulation, SCFA expression, regulation of mineral absorption, and immune response [134]. In recent years, studies on the gut-brain axis have shed light on the potential advantages of dietary fibers in NDD. The improvement of brain health by dietary fiber consumption appears to closely involve gut bacteria balance and SCFA production [105, 128]. For example, exopolysaccharides (EPS) produced by lactic acid bacteria during fermentation, show antioxidant effects that prevent neuronal cell death and mitochondrial dysfunction caused by varying levels of Aβ. EPS significantly reduces levels of caspase-7, caspase-8, caspase-9, CytC, and Bax in Aβ-treated SH-SY5Y cells, while concurrently increasing Bcl-2 levels [70]. A high-fiber diet (10% fibre) improved cognitive functions of HD mice, reduced the abundance of pathogenic bacteria, and decreased potentially pathogenic functional pathways in the gut microbe of HD mice [133]. Conversely, a long-term fiber-deficient diet (15 weeks) caused significant cognitive deficits in mice, including impairments in object location memory and temporal order memory, and difficulties in performing daily living activities. The fiber-deficient diet can damage the hippocampal synaptic structure and cause gut dysbiosis followed by a reduction in SCFA levels [135]. Table 1 summarizes the benefits of dietary fibers in NDD, categorizing them into three main groups: resistant oligosaccharides, non-starch oligosaccharides, and resistant starch [132].

**Table 1:** Common dietary fibers, sources, and their benefits against neurodegenerative diseases (NDDs)

Dietary fiber	Sources	Benefits against NDDs	References
*Resistant oligosaccharides*			
Fructo-oligosaccharides	Chicories, onions, leeks, garlics, tomatoes, bananas	-Decrease neuroinflammation -Improve cognition -Restore learning ability and memory -Regulate gut dysbiosis -Decrease intestinal inflammation -Prevent chronic stress	[134, 136, 137]
Galacto-oligosaccharides	Lentils, chickpeas, beans, human milk	-Decrease neuroinflammation -Improve cognition -Restore learning ability and memory -Regulate gut dysbiosis -Decrease intestinal inflammation	[134, 136, 138]
Inulin	Chicories, onions, leeks, garlics, tomatoes, bananas, citruses	-Antineuroinflammation -Reduces NDD risk -Enhances gut metabolism -Increases shortchain fatty acids (SCFA)	[134, 139]
*Resistant starch*			
Resistant starch	Grains, cereals, potatoes, legumes, seeds, nuts, bananas	-Improves brain function -Increases SCFA production -Regulates gut dysbiosis -Anti-inflammation -Protects against neuronal degradation	[134, 140]
*Non-starch polysaccharides*			
β-Glucan	Oats, barleys, seaweeds, mushrooms	-Improves cognition -Delays amyloid β plaque aggregation -Antineuroinflammation -Alters gut metabolites -Balances gut homeostasis -Decreases neuroinflammation	[95, 141, 142]
Pectin	Fruits and vegetables (sweet pepper, lemon, ginseng…)	-Neuroprotection against oxidative stress -Anti-inflammation -Enhances SCFA production -Modulates gut microbe -Reduces reactive oxygen species level	[87, 142, 143]
Arabinoxylans	Maizes, rice, wheats, other cereals	-Neuroprotection against oxidative stress -Anti-inflammation -Enhance SCFA production -Modulate gut microbe -Improve cognition -Regulate mitochondrial dysfunction	[65, 142]

##### Resistant oligosaccharides

Resistant oligosaccharides stand out as excellent dietary fibers and as prebiotics [134]. Notable examples include inulin, fructo-oligosaccharides (FOS), and galacto-oligosaccharides (GOS), which serve as prebiotics by promoting the growth of non-pathogenic bacteria such as *Bifidobacteria* and *Lactobacilli* [144]. FOS and GOS can directly regulate gut dysbiosis induced by chronic stress, leading to an increased ratio of *Bacteroidetes* to *Firmicutes* [96, 136]. They also facilitate the production of SCFAs in the colon, thereby reducing neuroinflammation and improving cognition function [105, 134]. These effects are attributed to the upregulation of the pro-survival pathway PI3K/Akt [136, 137]. Meanwhile, GOS can alleviate activation of microglia, such as lowering expression of Iba1, cytokines, and other inflammatory genes [138]. Inulin has been found to decline gut dysbiosis by promoting the growth of SCFA-producing bacteria such as *Blautia*, *Anaerostipes*, and *Bifidobacterium*. This leads to a reduced risk of AD in the APOE4 mouse model [139].

##### Resistant starch

Like the resistant oligosaccharides group, resistant starch exerts neuroprotection through its ability to maintain gut microbiome homeostasis [134]. Specifically, it promotes beneficial changes in gut composition such as increasing *Prevotella* or *Butyrivibrio*, which are closely linked to PD. Resistant starch can enhance certain brain functions in aged rodents, including glucose sensing and motor coordination [140]. However, the detailed pathways through which it exerts the neuroprotective effects are still to be elucidated.

##### Non-starch polysaccharides

Non-starch polysaccharides, functioning as prebiotics, communicate with the brain via the gut-brain axis. For instance, β-glucans exert anti-inflammatory properties by increasing the level of anti-inflammatory cytokine IL-10 and inhibiting the levels of pro-inflammatory cytokines IL-6 and TNF-α. The β-glucans can improve temporary memory and cognition by enhancing the expression of BDNF and PSD95 [141, 142]. Additionally, non-starch polysaccharides, such as pectin and arabinoxylan, exhibit novel neuroprotective effects based on their antioxidant activities. Pectin, for instance, may protect neuronal cells against oxidative stress stimuli by regulating phosphorylation of ERK1/2/Akt. This regulation leads to increased neuronal cell viability, reduced ROS levels, and reversed mitochondrial dysfunction [87, 143]. In a diabetic rat model of oxidative stress, arabinoxylan improves cognition by upregulating the activity of antioxidant enzymes GSH, SOD, pro-survival protein Akt, as well as neurotransmitters (dopamine, serotonin, acetylcholine). Arabinoxylan also reduces the expression of Aβ_1–42_, NF-κB, and cytokines IL-1β and TNF-α [65, 142]. These findings underscore the exciting potential of non-starch polysaccharides as a novel nootropic food ingredient suitable as a therapeutic intervention for managing NDD.

### Secondary nootropic metabolites

Secondary nootropic metabolites are organic compounds produced by organisms that are not directly required for basic growth, development, and reproduction. Instead, they possess specialized roles such as defense and signaling. Common secondary metabolites take the form of phytochemicals such as carotenoids, terpenoids, phytosterols, and polyphenols. These compounds are typically found in fruits, vegetables, roots, flowers, and plant-based food sources such as teas, marinades, herbal and whole foods [1, 10, 145–148]. These secondary metabolites have shown promising benefits for improving brain health, including managing and preventing NDDs. A summary of the outstanding advantages of well-known phytochemicals is provided in Table 2.

**Table 2:** Some common phytochemicals, sources, and their benefits for NDDs

Phytochemicals	Sources	Benefits for NDDs	References
*Carotenoids*			
β-Carotene	Yellow and orange fruits and vegetables e.g. carrots, orange	Neuroprotection against oxidative stress -Anti-inflammation -Stimulate DNA repair	[149, 150]
Lycopene	Yellow and red fruits and vegetables	-Neuroprotection against oxidative stress -Anti-inflammation -Reduces mitochondrial dysfunction -Prevents misfolded protein	[59, 151, 152]
Lutein	Green leafy vegetables (spinach, broccoli, kale…)	-Neuroprotection against oxidative stress -Anti-inflammation -Improves healthy aged brain -Regulates mitochondrial respiration during neurodevelopment	[153, 154]
*Polyphenols*			
Anthocyanins	Purple, red, or blue-colored fruits, vegetables, and rice (black rice, blueberries, grape…)	-Neuroprotection against oxidative stress -Antineuroinflammation -Improve healthy aged brain -Enhance cognitive function -Prevent misfolded protein	[1, 32, 52, 61, 155]
Resveratrol	Red grape, blackberries…	-Neuroprotection against oxidative stress -Antioxidative stress -Anti-inflammation -Prevents mitochondrial dysfunction -Improves memory	[10, 156, 157]
Curcumin	Curcuma (turmeric)	-Neuroprotection against oxidative stress -Anti-inflammation -Prevents mitochondrial dysfunction	[147, 158]
(-) Epigallocatechin-3-gallate	Green tea; apple skin, plum, onion, hazelnut	-Enhances cognitive function -Prevents misfolded protein	[73, 94]
Genistein	Soybean	-Improves cognitive function -Neuroprotective	[10, 94, 159]
Luteolin	Celery, green pepper, chamomile	-Neuroprotection against oxidative stress -Anti-inflammation -Freeradical scavenging	[69, 160]
Phenolic acids (protocatechuic, ferulic, gallic…)	Coconut oil, onion, red wine, grape, pomegranate	-Neuroprotection against oxidative stress -Anti-inflammation -Improve healthy aged brain -Enhance cognitive function -Prevent misfolded protein	[68, 91, 161]
*Terpenoids*			
Monoterpenes (limonene, camphor, 8-cineole…)	Citrus fruit oil, lemongrass oil, red wine	-Neuroprotection against oxidative stress -Anti-inflammation Psychoactive properties (relaxing, less anxious)	[68, 162–164]
Sesquiterpenes (β-caryophyllene, humulene…)	Thyme oil, wild basil oil, cannabi	-Neuroprotection against oxidative stress -Anti-inflammation -Enhance memory -Antidementia -Psychoactive properties (relaxing, less anxious…)	[165–167]

#### Polyphenols and their derivatives

Polyphenols are phytochemicals that have one or more phenolic rings esterified with hydroxyl groups, and are widely found in fruits, vegetables, and grains. Polyphenol compounds are characterized by their high antioxidant activity [168, 169]. These antioxidants demonstrate neuroprotective properties against oxidative stress by effectively scavenging ROS or regulating related enzymes. Their antioxidant activities are correlated with the modulation of oxidative enzymes such as NADPH oxidase in mouse neurons and SH-SY5Y cells. Meanwhile, they boost levels of antioxidant enzymes such as BDNF, heme oxygenase 1, or NADPH Quinone oxidoreductase 1 [25, 90]. They can block the activation of TLR-4 caused by LPS and inhibit neuro-inflammatory pathways [1, 10, 57] (Fig. 2). Particularly, anthocyanins can reduce the production of NO, IL-6, and TNF-α, as well as inhibiting the activity of inflammatory enzymes such as iNOS and COX-2 [60, 61].

Besides the strong effects against oxidative stress and neuroinflammation, in vitro and in vivo studies showed that polyphenol compounds can protect against NDDs through other mechanisms. For example, anthocyanins can lower Ca^2+^ elevation induced by exposure to glutamate or kainic acid and reduce Aβ deposition [32, 52]. Polyphenols such as anthocyanins and chrysoeriol derived from lutein can mitigate mitochondrial dysfunction by decreasing the levels of pro-apoptotic proteins such as JNK, p53, and p38 mitogen-activated protein kinase (p38-MAPK). Furthermore, EGCG in tea polyphenols can stimulate the expression of genes for pro-survival proteins PI3K and Bcl-2. These effects of polyphenols appear to be closely linked to the activation of Sirtuin-1 (SIRT1) [1, 86, 90]. Notably, certain polyphenols like EGCG or curcumin can form hydrogen bonds with DNA methyl transferase or histone deacetylases, which subsequently reduces the methylation level of SIRT1, thereby activating its expression [22, 170]. Flavonols and anthocyanins can alter histone modifications of genes, preventing the production of proteins in the NDD pathways including NF-κB, SIRT1, and MAPK (Fig. 2) [61, 147, 171]. These findings suggest potential use of polyphenols in the treatment of NDD. Curcumin treatment (160 mg/kg per day for 2 weeks) mitigates movement disorders in PD mice or rats [172, 173].

In addition, the indirect effect of polyphenols on NDD primary pathways through the gut-brain axis is equally important. Acting as prebiotics, polyphenols promote beneficial microbes and suppress harmful ones, thereby contributing to the gut flora (microbial) balance in the colon [168]. Upon fermentation by the gut microbiota, polyphenols are converted into small fragments or monomers such as phenolic acids, hydroxybenzoic acids, and hydroxycinnamic acids. These metabolites can be easily absorbed and delivered to brain cells to improve brain health. Small polyphenols like flavone luteolin or EGCG and their metabolites can cross the BBB and directly exert their neuroprotective effects as demonstrated in both in vitro studies and in animal models of AD [10, 69, 73]. Finally, resveratrol and flavonoids possess anti-microbial properties that may protect cells from infection and enhance the immune system including that in the brain [147, 171, 174].

It is important to note that the mechanisms and effects described in this section are derived from in vitro or preclinical studies. While these findings provide valuable foundational insights, their applicability to humans is limited due to challenges such as low bioavailability, BBB permeability, and physiological variability [175].

#### Terpenoids

Terpenoids, predominantly found in fruits, herbs, and spices, are natural arrangements of isoprene units (C5H8) with or without an oxygen moiety. They represent one of the largest groups of bioactive lipids [162, 176]. These compounds vary in size and are a challenge to analyze. Small volatile terpenes contain 5–15 carbon atoms such as hemiterpenes (C5), monoterpenes (C10), or sesquiterpenes (C15). Large nonvolatile terpenes can have 20–40 carbon atoms and include diterpenes (C20), triterpenes (C30), and tetraterpenes (C40) [171, 177]. However, large terpenoid compounds, such as cannabinoids derived from diterpenes (C20), phytosterols (C30), and carotenoids (C40), are often categorized differently due to their distinct structural properties and biological functions. These larger terpenoids are recognized for their potential therapeutic applications in NDDs and mental health. For example, cannabinoids have been studied for their neuroprotective and anti-inflammatory effects, while phytosterol and carotenoids are known for their antioxidant activities and epigenetic effects [67, 150, 178]. The role of carotenoids, as a subclass of terpenoids, will be explored further in the following section.

Small terpenoids have been reported to possess a wide range of medical properties that may benefit NDDs and mental health. Examples include anti-microbial, anti-inflammatory, antioxidant, and antidepressant activities [145, 146, 174]. Monoterpenes like camphor, 1,8-cineole, and α-thujone exhibit high antioxidant activity. They are capable of inhibiting enzymes responsible for inflammatory processes, such as 5-lipoxygenase, acetylcholinesterase, and xanthine oxidase [166]. These compounds also regulate key inflammatory pathways such as NF-κB and CCAAT-enhancer binding protein β, modulating the secretion levels of pro-inflammatory cytokines TNF-α and IL-6 [58]. Additionally, β-caryophyllene, a sesquiterpene, can bind and activate cannabinoid receptor 2, offering potential benefits in treating depressive disorders [145]. Most monoterpenes (C10) and sesquiterpenes (C15) are absorbed in the duodenum, the first part of the small intestine, or can even penetrate the skin. This enables them to confer their functional properties directly on the brain [146]. These small terpenoids also play a role in maintaining gut health by fighting harmful bacteria such as *Salmonella* and *Escherichia coli* [177].

#### Carotenoids

Many studies have demonstrated that carotenoids can reduce neuroinflammation and oxidative stress, two key NDD pathways. For example, mixtures of carotene and lutein can reduce ROS levels while increasing the expression of SOD, catalase, and NQO (NADPH dehydrogenase quinone) [150, 153]. Lycopene, another well-known carotenoid, can modulate the phosphorylation of MAPK and NF-κB and activate Nrf2 pathways, which offer protection against memory and cognition decline under exposure to LPS [59]. Additionally, carotenoids can regulate mitochondrial respiration via the PI3K/Akt pathway and modulate apoptotic proteins Bax/Bxl2, cytC, and caspase-3. They also influence the expression of synaptic proteins PSD95 and synaptophysin [152, 154]. In a rat model of AD induced by Aβ, lycopene improves neurogenesis by activating the Wnt/β-catenin pathway while downregulating the inhibitory protein GSK3β, thereby improving memory and spatial learning [151].

However, most carotenoids, like polyphenols and other phytochemicals with large molecular weight, have low bioavailability, which means that their direct impact on the brain is limited. Their influence is significantly mediated through interactions with the gut microbiome [177]. During digestion in the colon, these compounds engage in long-term interactions with gut microbes, contributing to homeostasis and improving brain health via the gut-brain axis [148, 168]. Carotenoids have high antioxidant and anti-inflammatory properties, which benefit the health of the gut microbiota. They enhance immune function and reduce intestinal inflammation by promoting the formation of immunoglobulin A. In addition, small amounts of carotenoids are broken down by beta-carotene oxygenase 1 into vitamin A, which supports brain structure and neuroplasticity [179].

## Nutrient supplements and dietary patterns rich in nootropic compounds for NDDs

Nutrient supplementation or diets rich in nootropic compounds such as PUFAs, essential amino acids, dietary fibers, polyphenols, and other phytochemicals, hold significant potential for therapeutic intervention of NDDs and mediating brain health outcomes [29, 30]. While in vitro and in vivo studies have suggested molecular mechanisms for their protective effects against NDDs, further confirmation of their impact on human health or NDD patients is necessary through clinical trials or observational studies [180].

### Individual or combined nootropic supplements

Randomized, double-blind, placebo-controlled clinical studies have linked specific individual or combined nutrients to brain health and mitigation of NDDs [180]. For example, PD patients treated with DHA and EPA capsules (800 mg/day DHA and 290 mg/day EPA) for 6 months experienced a 50% reduction in depressive symptoms, compared to only 25% in the placebo group [181]. In addition, supplementation with algal DHA (900 mg/day for 6 months) improved memory and learning abilities in older adults with age-related cognitive decline [182]. Similarly, co-supplementation of n-3 PUFA from flaxseed oil and vitamin E (1000 mg/day and 400 UI/day, respectively, for 12 weeks) significantly increased Unified Parkinson’s Disease Rating Scale (UPDRS) scores in PD patients. The treatment also changed metabolic status markers, including increased total antioxidant capacity (TAC) and GSH concentrations in the serum. This suggests that the intervention may exert these effects by reducing neuroinflammation and oxidative stress [183]. However, some studies have reported negligible effects of n-3 PUFA treatment (for 16 weeks) on cognition and mood of AD patients. The inconsistency highlights the need for further research with longer durations, larger sample sizes, varied doses, or the consideration of dementia subtypes. Additionally, the interaction of PUFAs with other compounds in whole food products or general diets should also be considered in future studies [3, 184].

Recent cohort studies have revealed a strong association between amino acid metabolism and low marker signals of NDD. For example, decreases in the ratio of methionine to homocysteine, the ratio of *L*-arginine to nitric oxide, and tryptophan levels in AD patients or people with dementia are linked to reduced gray matter volume loss and the presence of *APOE*-ε4 allele [113, 119, 185, 186]. Consequently, the amino acid profile may serve as a promising diagnostic tool for NDDs and a monitoring biomarker for NDD treatment [187]. Clinical studies have shown the potential benefits of amino acids in various aspects of mental health and cognitive function. For instance, tryptophan-enriched drinks improves mood, emotional stability, and cognitive functions, such as improved happiness before sleep, slower response to negative words, and sustained attention in middle-aged women [188]. Similarly, *L*-arginine treatment can counteract cognitive impairment in hypertensive adults by reducing oxidative stress in the mitochondria of endothelial cells [189]. A 12-week intervention with a mixture of seven amino acids (6 g/day) resulted in significant improvements in cognitive function, social interaction, and psychological health among adults aged 55 and older [190]. Despite these promising findings, clinical trials investigating amino acid supplementation for NDDs remain limited. Recent research has indicated the potential of *L*-lysine in enhancing electroencephalography patterns, reducing seizure frequency, and improving abnormal behavior in individuals with neurodevelopmental disorders linked to *GRIN* genes. The *GRIN*-gene-related disorder involves the mutation of NMDAR [191].

Similar limitations can be observed in clinical trials investigating the impact of dietary fibers and secondary metabolites on NDD. While there is emerging evidence of their benefits on brain health and in NDD, the research remains preliminary. Data on high-fiber-diet-related metabolites in elderly patients with type 2 diabetes mellitus (T2DM) suggest that these diets may reduce neurodegenerative symptoms with obesity. In T2DM patients with neurodegenerative symptoms, high-fiber diets influence autophagic homeostasis and NF-κB signaling pathways in the hippocampus through binding of the metabolite acetamidobenzoic acid to SPEG [192]. In patients with PD, a 10-day intervention with different dietary fibers led to promising changes in gut microbiota, SCFA, inflammation, and expression of neurofilament light chain (NfL), a marker associated with NDD. In particular, resistant starch (raw potato starch) appears to be more effective than inulin in producing butyrate and lowering NfL levels [193].

Long-term intake of polyphenols such as resveratrol (200 mg/day for 26 weeks) and curcumin (180 mg/day for 18 months) can significantly enhance memory, attention, and hippocampal functional connectivity in healthy elderly individuals. These compounds may ameliorate misfolded protein aggregation, neuroinflammation, and high glucose levels linked to oxidative stress during brain aging [156, 194]. Similarly, the elderly with mild dementia impairment receiving carotenoid treatment, including lutein (10 mg/day) and zeaxanthin (2 mg/day) for 1 year, experienced improvements in attention and cognition [195].

However, the benefits of these interventions for NDDs are not always consistent and require further investigation. For instance, catechins (227 mg/day) and theanine (42 mg/day) from green tea powder, when taken for 3 months, may enhance general cognitive impairment and short-term memory. However, results are not significant across different stages of cognitive deficit [196]. In PD patients, curcumin (2 g/day for 12 months) reduces the aggregation of phosphorylated α-synuclein and improves some clinical scores such as UPDRS [197]. In contrast, another study with a lower dose (80 mg/day for 9 months) revealed no significant improvement in PD symptoms. The overall trend of UPDRS in motor examination parameters differed between the curcumin and placebo groups, but no significant differences were observed at each time point [198]. This inconsistency may stem from many factors such as disease severity of NDDs and duration of the treatment. Shorter disease durations with less damaged neurons may be associated with better improvement by the treatment [197, 198]. The bioavailability, BBB permeability and variable physiological concentrations of compounds may also play an important role [175]. For example, after 6 months of curcumin supplementation at 4 g/day, the plasma levels of curcumin in mild-to-moderate AD patients were notably low (only 7.32 ng/ml). This may explain the lack of significant cognitive improvement and the minimal effects on cerebrospinal fluid biomarkers such as Aβ_1–42_, t-tau, or p-tau_181_ [78].

### Effect of dietary patterns

Cohort studies have revealed various impacts of dietary patterns on brain health and NDDs. Diets rich in PUFAs are positively associated with enhanced human brain health, indicated by increased gray matter volumes and improved brain glucose metabolism [199]. In AD patients undergoing treatment with acetylcholinesterase inhibitors, lower serum levels of n-3 PUFA, particularly DHA, are correlated with a higher risk of cognitive decline [200]. High-fiber diets also mitigate neurodegenerative symptoms in obese individuals, likely through modulation of autophagy and NF-κB pathways [192]. Furthermore, diets with more complex dietary patterns have shown similar results. For example, a higher intake of vitamin B12 and vitamin D, particularly when combined with n-3 PUFAs or zinc, can decrease the Aβ load. Concurrently, elevated consumption of β-carotene/folate or antioxidants/fibers is linked with increased glucose metabolism in the brain, a marker of neuronal activity [199, 201]. These nutrient effects are notably pronounced in *APOE4* carriers and those with a family history of AD. The findings suggest that specific dietary choices may influence the AD risk [201]. In PD patients, plasma levels of PUFAs such as ALA, LA, and ARA are lower than expected, despite similar dietary intake as controls. This discrepancy suggests a potential alteration in lipid metabolism due to factors such as neuroinflammation and impaired absorption [202, 203]. However, despite the role of PUFAs in PD, their effects are multifaceted, and the benefits of supplementation remain uncertain, warranting further research. Specifically, plasma levels of ALA and LA are inversely correlated with motor severity, while DHA and ARA plasma levels are positively associated with non-motor symptoms in PD [203].

Various dietary patterns, such as the Mediterranean diet (MedDiet), ketogenic diet, and other health-focused dietary guidelines, have been explored as potential for NDD intervention. The MedDiet, known for its high intake of fruits, vegetables, whole grains, fish, olive oil, and red wine, is rich in fibers, PUFAs, polyphenols, carotenoids, and terpenoids [204]. The DASH diet (Dietary Approaches to Stop Hypertension), similar to MedDiet, focuses on fruits, vegetables, and whole grains but emphasizes low sodium intake. Combining elements of these two diets, the Mediterranean-DASH Intervention for Neurodegenerative Delay (MIND) adds specific recommendations for green vegetables and berries, which are independently associated with neuroprotection [205–208]. Adherence to these dietary patterns is evaluated by the frequency of healthy component consumption and the limitation of unhealthy components (such as red meat, full-fat dairy products, sweets, and fast food). Strong adherence to the MedDiet or MIND diets has been significantly correlated with healthy cognitive aging, reduced risks of dementia, and lower levels of NDD pathology [205, 208–210]. In older adults (aged 63–76), higher MedDiet or MIND scores are associated with healthier brain structural markers, such as larger gray matter or hippocampus volume, and thicker cortex, which result in improved memory performance [206, 211]. Similar benefits have been observed with adherence to other high-quality diets, such as Dutch dietary guidelines, which emphasize a high intake of vegetables, fruit, whole grains, legumes, nuts, dairy, fish, and tea [212]. These dietary patterns have also been positively correlated with better cognitive performance, particularly in memory, as demonstrated in neuropsychological tests [208–210]. In addition, adherence to these diets correlates with a significant reduction in pathology markers such as Aβ, phosphorylated tau181, and *APOE4* status [205, 211]. Interestingly, these effects seem to be independent of age and genetic risk factors (such as *APOE4*), further indicating the possible roles of nootropic diets in mitigating NDD risks [206, 209, 210].

The low-carbohydrate or ketogenic diet (keto diet) also shows promise, particularly in alleviating anxiety and depression symptoms in PD patients. A 24-week intervention with keto diets (78% fats, 17% protein, and 3%–4% carbohydrates) resulted in reduced scores on common symptom scales of PD, including the CESD (Center for Epidemiologic Studies Depression) scale and the Parkinson Anxiety Scale [213]. The Mediterranean ketogenic diet (MMKD), which combines the ketogenic approach with the MedDiet, introduces carbohydrate restriction alongside limited whole grains with an increased focus on fruits and vegetables within set limits. The MMKD also emphasizes low-fat protein sources, such as fish and lean meats, as well as healthy fats such as extra virgin olive oil. A 6-week MMKD consumption (5%–10% carbohydrate, 30% protein, and 60%–65% fat) increased cerebral ketone body uptake (measured by ^11^C-acetoacetate positron emission tomography scans). This change was associated with increased Aβ_42_ and decreased tau levels in cerebrospinal fluid, and improved memory performance [77]. Additionally, MMKD may modulate specific gut fungal signatures in patients with mild cognitive impairment, such as increased *Agaricus* and *Mrakia,* as well as decreased *Saccharomyces* and *Claviceps*. Interestingly, MMKD intervention can influence the correlation between gut fungal species and AD biomarkers such as p-tau. During MMKD treatment, the patients showed a negative correlation between p-tau levels in cerebrospinal fluids and proportions of *Aspergillus* and *Cladosporium* [214].

## Development of nootropic food products for NDDs

### Demand for nootropic products for NDDs

As discussed, in vitro, in vivo and observational studies have consistently shown that certain nutrients such as antioxidants, PUFAs, and fibers are associated with lower incidences of cognitive decline, dementia, and NDDs. Interventional trials focusing on nutritional supplementation, while showing some benefits on NDD outcomes, often report effects that are modest, not statistically significant, and inconsistent [180, 197, 198, 203, 215]. One potential explanation for this inconsistency is the failure to consider how these supplements interact within the broader context of an individual’s overall diet and gut metabolism. For example, a higher intake of omega-3-rich foods might correlate with a lower consumption of less healthy options, such as certain types of red meat, which could contribute to the observed benefits [180]. This highlights the need to investigate the effect of diet on NDDs by focusing on holistic dietary patterns rather than individual nutrients in isolation [215]. In recent studies, traditional Mediterranean or ketogenic diets along with their modified versions hold promise for supporting cognitive function and reducing NDD risks. However, these diets often require strict adherence and significant changes in eating habits, which can be challenging for individuals, particularly those already experiencing NDDs and other neurological disorders. Additionally, the studies referenced used various methods to assess dietary exposure, including diet history and food frequency questionnaires. This diversity in assessment techniques could have introduced inconsistencies in the accuracy of the data, as self-reported dietary intake does not always correspond to actual biological nutritional status [203].

Considering these challenges, there is growing interest in developing food-based nootropic products that combine multiple nutrients and bioactive compounds, such as those derived from natural extracts. Besides, neurodegeneration is a multifactorial process involving excitotoxicity, neuroinflammation, oxidative stress, mitochondrial malfunction, and misfolded protein load. Integrative approaches that address these interconnected processes are required to provide more effective outcomes in combating NDDs. Specifically, food products should utilize diverse nutritional ingredients and synergistically target these various pathological pathways [3, 94, 216]. These nootropic products are particularly appealing because they can be seamlessly integrated into daily diets. This makes them more accessible and easier to adhere to than strict dietary regimens. By leveraging the benefits of nootropic food components, nootropic products can be developed as effective interventions to reduce that risk of or manage NDD. These products can be easily integrated into healthy dietary patterns to support brain health.

### Challenges and future directions

**Fig. 4:**
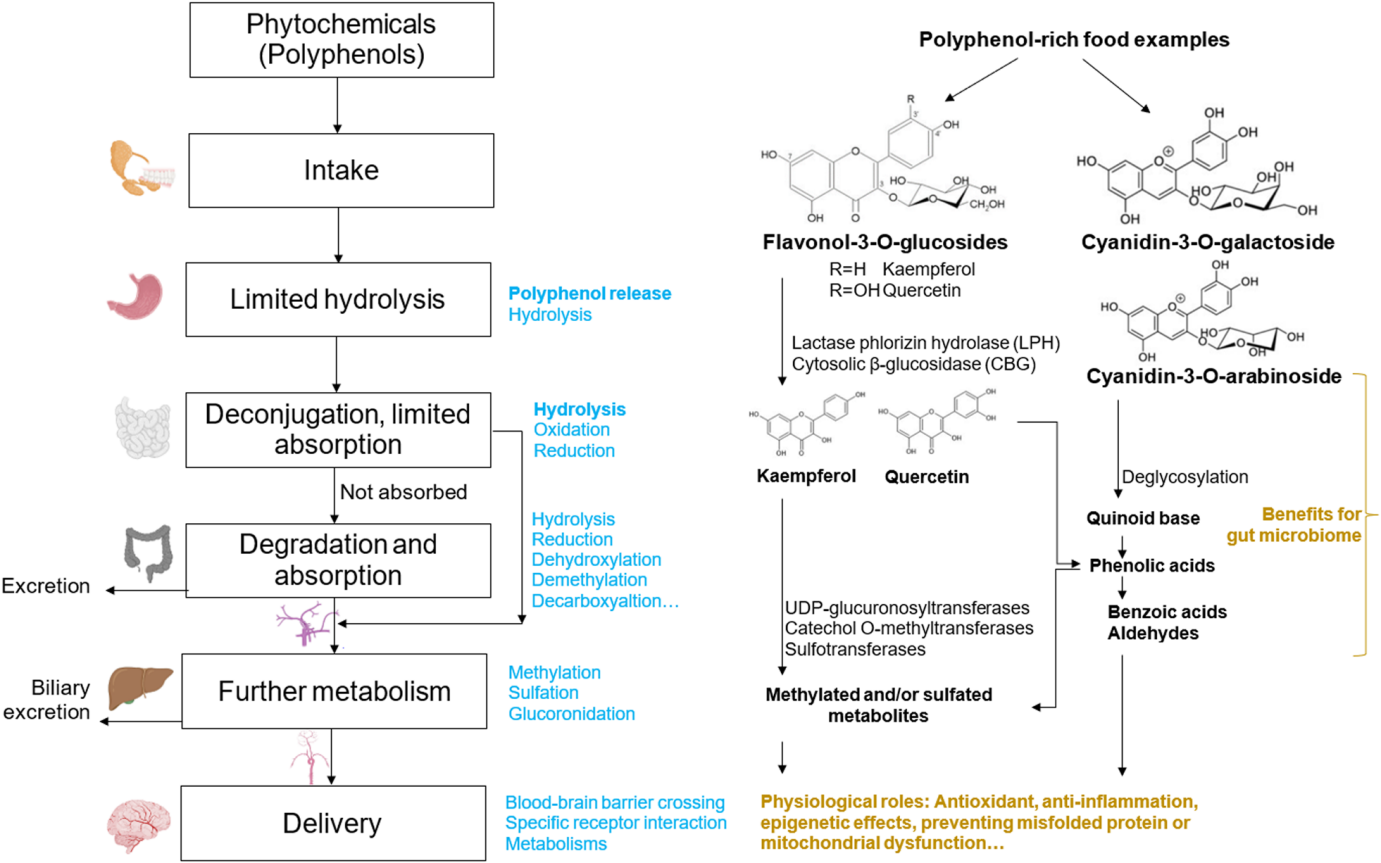
The major digestive process and absorption of polyphenol-rich ingredients

Food-based nootropic products face significant challenges, particularly concerning the bioavailability of active ingredients. The bioavailability can vary widely when delivered through food matrices under physiological conditions, often leading to inconsistent therapeutic outcomes [94]. Certain small and non-polar phytochemicals such as monoterpenes or sesquiterpenes are efficiently absorbed in the small intestine [146]. Meanwhile, larger phytochemicals may require enzymatic processing in the small intestine, such as lactase phlorizin hydrolase or cytosolic β-glucosidase, to facilitate their absorption, metabolism, and eventual delivery to the brain [177, 217, 218] (Fig. 4).

Some phytochemical compounds, including curcumin, resveratrol, quercetin, EGCG, isoflavones, and phenolic acids, may directly cross the BBB and exert effects on brain functions at low nanomolar concentrations [1, 73, 156]. For example, it has been reported that polyphenols can be neuroprotective at low nanomolar levels by binding to the laminin receptor of neuronal cells. At levels as low as 0.01–10 nmol/L, resveratrol and its metabolite glucuronide prevented neuronal cell death induced by serum starvation via cAMP-mediated signaling pathways [219]. However, the low bioavailability of most of these compounds significantly limits their efficacy. Many are not effectively digested in the small intestine. Instead, they are often degraded by gut microbiota in the colon, further reducing their potential impact. To address these challenges, emerging strategies such as nanoencapsulation and advanced delivery systems have been developed to enhance bioavailability and improve targeting mechanisms [28, 94, 216]. For instance, grafting resveratrol and oligomeric proanthocyanidin with hyaluronic acid and peptide B6 into nanoparticles enhances their permeability into BBB to reduce ROS and inflammation in the brains of AD rats [220]. Similarly, nanoparticle form of resveratrol using poly(lactic-co-glycolic acid) nanoparticles conjugated with lactoferrin has enabled the high diffusion distribution in the brains of PD mice, yielding neuroprotective effects [221]. These promising results highlight the potential of novel delivery approaches; however, they require further investigation in further focused studies and validation through rigorous, well-designed clinical trials.

Additionally, the complex interactions between different nutrients or bioactive compounds can result in unpredictable effects, which may further diminish their overall efficacy. For example, the dietary fats may interfere with the therapeutic effects of PUFA or hinder the absorption of bioactive compounds. Vitamins and other nutrients can contribute to the significant variability observed in clinical outcomes [3, 202]. Optimizing nootropic formulations, including exploring novel sources of nootropic compound mixture, will be crucial to maximize their therapeutic potential [3, 10, 31]. For example, milk fat globule membrane (MFGM) has gained high attention for its potential benefits for cognitive and mental health. MFGM is a rich source of bioactive polar lipids, proteins, and glycans, which play vital roles in supporting synaptic plasticity, reducing neuroinflammation, maintaining the integrity of the BBB, and modulating gut-brain communication. MFGM supplementation can enhance cognitive functions, particularly in early life stages, and may offer neuroprotective benefits in aging populations [222–224]. Integrating MFGM with other bioactive compounds, such as DHA and α-lactalbumin rich in essential amino acids, into infant milk formulas, could offer synergistic effects, further enhancing its therapeutic potential [225, 226].

The long-term safety and efficacy of many nootropic ingredients and nootropic foods, including MFGM, remain under-researched in the context of NDD, requiring more robust clinical evidence. Future studies should examine the combined effects of multiple dietary factors and the suitable matrix foods for nootropic ingredients to achieve the high bio-accessibility [227, 228]. The neuroprotective efficacy of nootropic foods in relationship with additional lifestyle elements like physical activity should be considered as they are known to influence both dietary behavior and cognitive function [94, 215].

Advanced techniques such as lipidomics and food metabolomics should be integrated to develop and validate neuroprotective or nootropic foods. These tools can provide a comprehensive view of cellular processes by analyzing lipids and metabolites, which are critical components of neuronal function and regulation [157, 229]. They mainly involve advanced gas chromatography (GC), liquid chromatography (LC) coupled with mass spectrometry (MS), and other detectors like flame ionization detectors (FID). These techniques can help identify key metabolic pathways and biomarkers closely related to food factors during treatments and reveal molecular mechanisms underlying the inconsistencies observed in clinical trials. For example, untargeted LC–MS has revealed that oxy-resveratrol protects SH-SY5Y cells against rotenone cytotoxicity mainly through modulating the dopamine biosynthesis pathway. The pathway was uncovered by the determination of increased levels of metabolites such as phenylalanine, 7,8-dihydrobioprotein, tyrosine, and dopamine with oxy-resveratrol treatment [157]. Similarly, GC-FID has been instrumental in confirming altered fatty acid metabolism in PD patients by revealing a mismatch between plasma PUFA levels and dietary intake, which suggests malabsorption in these individuals [203]. By leveraging these advanced techniques, researchers can refine nootropic products, ensuring more consistent and effective results in the prevention and management of NDDs.

## Conclusions

Neuroprotective and nootropic foods are known for their benefits in cognitive enhancement and protection against neurodegeneration. These foods are rich in bioactive compounds such as natural polyphenols, omega-3 fatty acids, and fibers. They are believed to exert neuroprotective effects through various mechanisms such as reducing oxidative stress and inflammation, modulating neuronal signaling pathways, and supporting gut balance.

Despite their potential, the development and validation of nootropic foods face significant hurdles. A major challenge is the difficulty in accurately assessing and validating their effects, given the complex interactions between different nutrients and individual variations in diet and genetics. Current research often focuses on isolated specific nutrients, potentially overlooking the combined effects that occur within whole food products or whole dietary patterns. Additionally, enhancing the bioavailability of bioactive compounds is crucial. Modern techniques such as nano-emulsion and encapsulation should be considered to improve the efficacy of these compounds.

Optimization of nootropic formulations within whole food products is an important direction for future research. Besides, incorporating advanced analytical methods like lipidomics and metabolomics could advance understanding of how dietary patterns or nootropic food products influence brain health and NDDs and provide guidance for nootropic food innovations. These approaches may contribute to bridging the gap between laboratory findings and clinical applications, offering a clearer picture of the potential therapeutic benefits of these foods.

## Supplementary Information

**Additional file 1**. **Figure S1.** Flow chart of study selection in this review.

## Data Availability

Not applicable.

## Abbreviations

AD: Alzheimer’s disease
ALA: α-Linolenic acid
ALS: Amyotrophic lateral sclerosis
APP: Amyloid precursor protein
ARA: Arachidonic acid
Aβ: Amyloid beta
BBB: Blood–brain barrier
BDNF: Brain-derived neurotrophic factor
COX-2: Cyclooxygenase 2
DHA: Docosahexaenoic acid
EGCG: Epigallocatechin-3-gallate
EPA: Eicosapentaenoic acid
FOS: Fructooligosaccharides
GC: Gas chromatography
GPCRs: G-protein-coupled receptors
IL: Interleukin
iNOS: Inducible nitric oxide synthase
LA: Linoleic acid
LC: Liquid chromatography
LPS: Lipopolysaccharide
LTB: Leukotrienes B
MAPK: Mitogen-activated protein kinase
NADPH: Nicotinamide adenine dinucleotide phosphate
NDD: Neurodegenerative diseases
NEP: Neprilysin
NF-κB: Nuclear factor kappa B
NMDAR: N-methyl D-aspartate receptor
NOX-2: NADPH oxidase 2
Nrf2: Nuclear factor erythroid 2-related factor 2
PD: Parkinson’s disease
PGE2: Prostaglandin E
PUFA: Polyunsaturated fatty acid
ROS: Reactive oxygen species
SCFA: Short-chain fatty acid
SIRT1: Sirtuin-1
ThB: Thromboxane
TLR-4: Toll-like receptor-4
TNF-α: Tumor necrosis factor-alpha
