# Supplementary figures and images for: Nootropic foods in neurodegenerative diseases: mechanisms, challenges, and future
Source: Transl Neurodegener. 2025 Apr 3;14:17. doi: 10.1186/s40035-025-00476-7 (PMC11967161; doi:10.1186/s40035-025-00476-7)

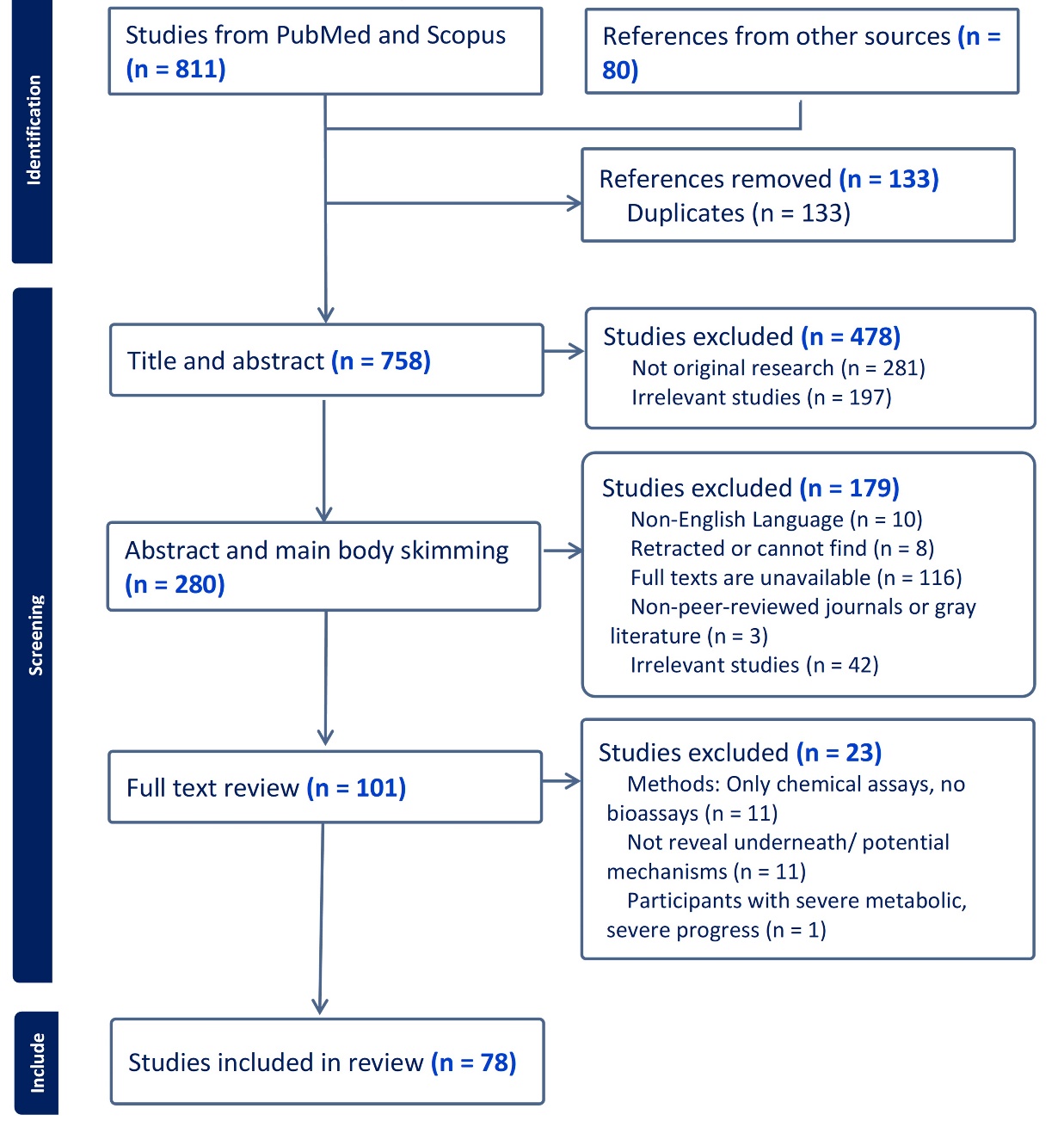


**Figure S1.** Flow chart of study selection in this review.

Supplement: Supplementary file 1 — Additional file 1. Figure S1. Flow chart of study selection in this review. [file 40035_2025_476_MOESM1_ESM.docx]
